# Vertical Bite Rehabilitation of Severely Worn Dentitions with Direct Composite Restorations: Clinical Performance up to 11 Years

**DOI:** 10.3390/jcm10081732

**Published:** 2021-04-16

**Authors:** Tobias T. Tauböck, Patrick R. Schmidlin, Thomas Attin

**Affiliations:** Department of Conservative and Preventive Dentistry, Center for Dental Medicine, University of Zurich, 8032 Zurich, Switzerland; patrick.schmidlin@zzm.uzh.ch (P.R.S.); thomas.attin@zzm.uzh.ch (T.A.)

**Keywords:** tooth wear, dental erosion, abrasion, resin composite restorations, occlusal vertical dimension

## Abstract

Our aim was to evaluate the clinical performance of direct composite restorations placed in patients with severely worn dentitions at an increased vertical dimension of occlusion, after up to 11 years. One hundred and sixty-four teeth in 13 patients with severely worn dentitions had been reconstructed with either microhybrid (first cohort; *n* = 59) or nanofilled (second cohort; *n* = 105) composite restorations at increased vertical dimension of occlusion using a wax-up-based template-aided placement technique. From the dental records, information about repair and replacement of restorations was obtained. Patients were clinically examined after a mean follow-up time of 10.7 years (first cohort) or 5.2 years (second cohort) using United States Public Health Service (USPHS) criteria. Subjective patient satisfaction was also recorded using visual analogue scales (VAS). The overall quality of the restorations was good with predominantly ‘Alpha’ and ‘Bravo’ scores, respectively. Nanofilled composite showed less surface degradation and better margin qualities than microhybrid composite. Of the 59 restored teeth in the first cohort, 13 restorations showed unfavorable events after 10.7 years, of which ten could be repaired. In the second cohort, 23 of 105 restorations showed unfavorable events, which could all be repaired. VAS scores revealed high patient satisfaction with the treatment approach. In conclusion, direct composite restorations placed at an increased vertical dimension of occlusion show good clinical long-term performance in patients with severe tooth wear.

## 1. Introduction

Tooth wear represents an irreversible, multifactorial, non-carious loss of dental hard tissues based on erosive, abrasive and/or attritional effects [1,2]. Epidemiological data suggest that tooth wear is becoming increasingly common, especially in younger populations, with prevalence and extent rising with age [3,4,5]. Apart from epidemiological concerns, tooth wear may manifest itself in severe forms and lead to tooth hypersensitivity, loss of vertical dimension of occlusion, esthetic impairment, and compromised oral health-related quality of life at the individual patient level [6,7].

Patients suffering from severe tooth wear require complex restorative care to compensate for the loss of tooth substance, particularly if the occlusal vertical dimension is affected. Traditional reconstructive concepts for the severely worn dentition basically comprise extensive prosthodontic treatment such as full or partial crown coverage of the majority of teeth [8,9,10]. Apart from being expensive and thus unaffordable for many patients, this conventional approach is highly invasive due to required tooth preparations sacrificing significant amounts of sound dental hard tissue. Therefore, a re-thinking in the restorative management of tooth wear toward minimum-intervention approaches has taken place, which rather focuses on conservative “additive” instead of conventional “subtractive” treatment strategies according to a dynamic restorative concept taking prophylactic and reparative considerations into account [11].

In particular, the use of direct resin composite materials allows for such conservative, non-invasive restorative treatment modalities. Moreover, resin composite restorations are relatively inexpensive, provide good overall esthetics as well as simple maintenance in the form of refurbishment and repair [12,13]. Numerous studies substantiate that small- to medium-sized intra-coronal composite restorations show good and reliable clinical long-term results in load-bearing posterior teeth [14,15,16]. Owing to improvements in material technology and clinical application techniques [17,18,19,20], the range of indications of direct composite restorations has been continuously extended over the years, covering many indications, which were originally reserved for indirect reconstructions only [21]. However, the use of resin-based composites to restore severely worn teeth and reconstruct the occlusion remains controversial, and clinical data are still scarce. Bartlett and Sundaram [22] reported high rates of fracture and retention loss when treating worn posterior teeth with direct, but also with indirect, resin composite restorations. More recent studies, in contrast, showed more favorable clinical performance of full rehabilitations with resin-based composite placed even at increased vertical dimension discrepancies [23,24,25,26,27].

In order to avoid freehand shaping of the direct composite restorations and ensure optimal adjustment of the vertical dimension of occlusion, a wax-up-based template-aided placement approach has been suggested and has proven to be easily implemented by general practitioners [28]. The 3- and 5.5-year clinical performance of template-aided posterior vertical bite composite reconstructions in severe tooth-wear patients has been previously reported [23,25]. However, observation periods of these studies still address the short- to medium-term performance of such restorations. The clinical long-term behavior of composite vertical bite reconstructions is as yet unknown, and, consequently, investigations with longer observation periods are highly needed.

Therefore, the purpose of the present investigation was to re-evaluate the original restorations of this first cohort of patients after a mean service time of 10 ⅔ years. In addition, a second cohort of patients with posterior vertical bite reconstructions made of nanofilled composite material, instead of the originally used microhybrid composite, was evaluated to identify possible material-related differences in the clinical performance of the vertical bite reconstructions. The subjective patient satisfaction with the vertical bite composite reconstructions was assessed using visual analogue scales (VAS).

## 2. Materials and Methods

### 2.1. Patients and Treatment

The investigation was approved by the local ethical committee of the Canton of Zurich (KEK-ZH-Nr. 2015-0059), and written informed consent was obtained from all participants. Of the six patients of the first cohort evaluated in the 5.5-year follow-up, five returned for the present follow-up examination. One patient had moved to another city and could not be located despite several attempts. The mean observation time of this first patient cohort with a total of 59 posterior restorations was 10.7 ± 0.4 years (range: 10.2–11.2 years) at recall (Table 1). In the second cohort, eight patients of our clinic who received posterior vertical bite reconstructions after an update of the clinical protocol with regard to the used composite material, adhesive system, and application technique were evaluated. Only patients with vertical bite reconstructions with a service time of at least three years were included. In the second cohort, a total of 105 posterior restorations were evaluated. The mean observation time of these restorations was 5.2 ± 1.4 years, with a range of 3.4–7.2 years (Table 2). In both cohorts, only patients with mainly erosion-induced tooth wear and no signs of temporomandibular disorders were included. Before restoration, elimination of the causative erosive factors was achieved in all patients. To this end, dietary control and/or medical and psychological treatment were provided where necessary, and patients were instructed on individual erosion prevention measures.

The restorative treatment has been previously described in detail [23]. Full-arch cast models were produced and mounted on a semi-adjustable articulator. A diagnostic wax-up of all teeth was then made in a balanced occlusion, providing the intended vertical bite reconstruction. The increase of the vertical dimension of occlusion amounted to 2–3 mm for all patients. Based on the wax-up, patients of the first cohort received a stabilization splint that was worn for four to six months to simulate the new vertical bite position prior to restoration. Due to the relatively low extent of increase of the vertical dimension of occlusion and the exclusion of patients with temporomandibular disorders, this splint pretreatment was no longer performed in patients of the second cohort [29].

To enable accurate transfer of the waxed-up occlusion from the articulator into the patient’s mouth, wax-up-based templates were fabricated. These transfer aids were either full-arch vacuum-formed matrix templates (first cohort) or short quadrant templates made of transparent acrylic and relined with transparent bite registration material (second cohort). The templates revealed a hollow space between the occlusal surface of the natural teeth in the oral cavity and the inner facet of the template, in the dimension of the wax-up. This hollow space was later filled with the composite material to build up the worn teeth and copy the wax-up. Both the front teeth and the most distally located teeth, which had not been waxed up previously, assured stable support of the templates. The most distal teeth were restored using a freehand build-up technique without template. The front teeth were reconstructed at a later appointment.

**Figure 1:**
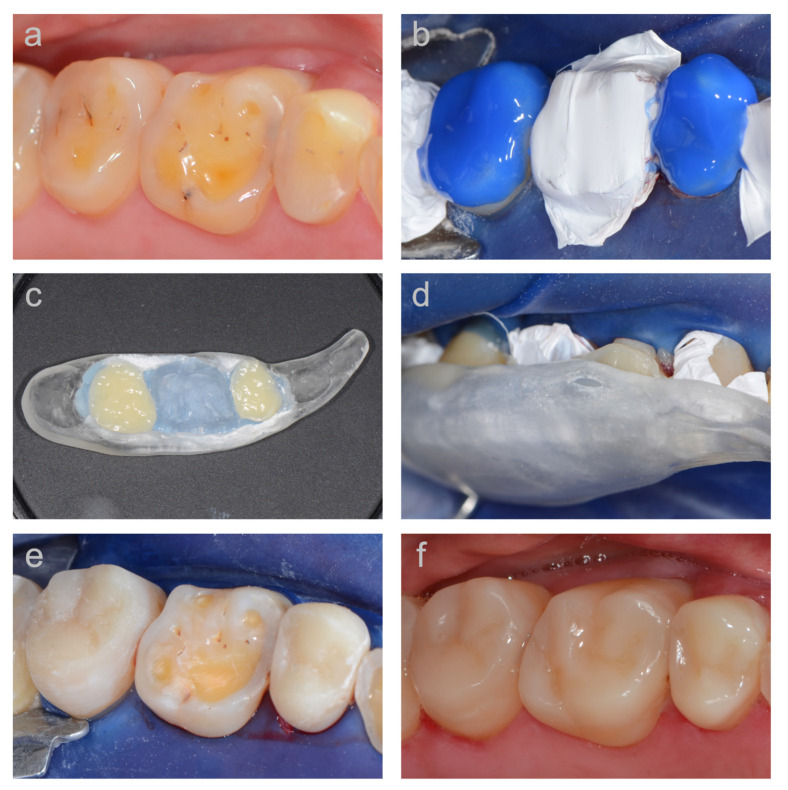
Step-by-step documentation of the TACS-technique (‘Template-Aided Composite Shaping’) used for the direct composite vertical bite reconstructions. (**a**) View of the eroded teeth before treatment; (**b**) adhesive pretreatment of every second tooth and isolation of the adjacent teeth with Teflon tape to avoid interproximal blocking with composite material; (**c**) detailed view of the wax-up-based short quadrant template made of transparent acrylic and relined with transparent bite registration material. The template is placed on a heating plate to preheat the composite inside the template; (**d**) template, filled with uncured composite, positioned on the tooth arch; (**e**) composite build-ups of teeth 15 and 17 after pre-finishing; (**f**) completely reconstructed occlusal surfaces of teeth 15–17 after finishing and polishing.

Before restoring the vertical dimension, all metallic or insufficient restorations were replaced with composite restorations. In the patients of the first cohort, the adhesive system Syntac Classic (Ivoclar Vivadent, Schaan, Liechtenstein) and the microhybrid composite material Tetric (Ivoclar Vivadent, Schaan, Liechtenstein) were used, while in the second cohort, the adhesive system Optibond FL (Kerr, Orange, CA, USA) and the nanofilled composite Filtek Supreme (3M, St Paul, MN, USA) were used, each according to the manufacturers’ instructions. The vertical bite reconstruction was performed under local anesthesia and rubber dam placement. Surfaces of existing intact composite restorations were sandblasted with aluminum oxide and silanized. After conditioning the tooth surfaces with the respective adhesive system, the occlusal relief of the worn teeth was reconstructed by using the wax-up-based template. A step-by-step documentation of this TACS-technique (‘Template-Aided Composite Shaping’) is provided in Figure 1.

During restorative treatment, every second tooth of each quadrant was first conditioned and built up. Either small strips of metal matrices were interproximally placed, or Teflon tape was used to insulate the adjacent teeth in order to avoid interproximal blocking with composite material, thus allowing interproximal passage with dental floss after the reconstruction. A small amount of composite material was applied onto the conditioned occlusal tooth surfaces. Then, the template was filled with composite and positioned on the tooth arch. In the second cohort, the uncured composite material inside the template was preheated for 5 min on a heating plate (Calset, AdDent, Danbury, CT, USA) to reduce composite viscosity and facilitate placement of the template without compromising physical properties of the composite material [30]. The composite was initially photoactivated only briefly through the template for 3–4 s. After careful removal of the template, excess composite material could be removed from the teeth using a scalpel, and the composite was subsequently fully light-cured for an additional 60 s. After finishing and polishing, the remaining teeth were restored in the same way, and the occlusion was carefully controlled. Following the posterior vertical bite reconstruction, the anterior teeth were restored with either resin composite or indirect ceramic restorations. Due to the different restoration types, the anterior teeth were not included in the follow-up examinations [23,25].

After restorative treatment, all patients received acrylic splints (night guards) to protect the restorations. Regular recall appointments were agreed based on an individual periodontal and cariologic risk assessment, with a maximum interval of six months.

### 2.2. Clinical Evaluation

Restorations still in place at the time of follow-up were evaluated according to modified United States Public Health Service (USPHS) criteria (Table 3) by two investigators independently, who were pre-calibrated at 94% reliability. Disagreements were resolved by discussion between the two investigators until consensus was achieved. When a restoration was lost, or had to be replaced during the observation period, it was defined as a failure. When a restoration needed repair, it was defined as survival. Restorations with no failure and without repair were defined as success.

### 2.3. Patient Satisfaction/Compliance

Patient-reported outcome measures (PROMs) were examined using visual analog scales (VAS). All patients were asked four questions about their satisfaction with the treatment in terms of functional parameters, acceptability, and cost-benefit ratio, and marked their response for each question on a 100-mm horizontal VAS. The response on each VAS was measured with a ruler to the nearest millimeter. Furthermore, patients were asked if they used the night guard as prescribed.

## 3. Results

### 3.1. First (Long-Term) Cohort

**Figure 2:**
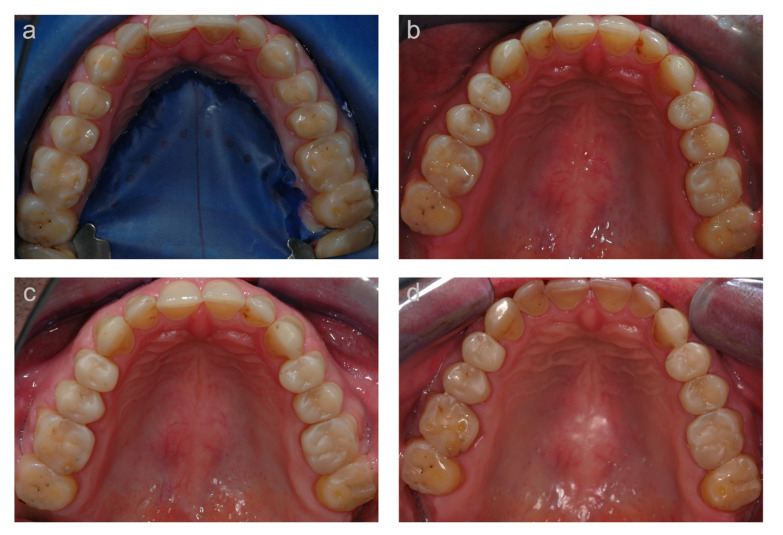
Example of a case (occlusal view of upper jaw) of the first cohort. (**a**) Clinical situation at the reconstructive appointment before treatment; (**b**) clinical situation at the 2 year, (**c**) 5-year, and (**d**) 10-year follow-up.

The results of the clinical evaluation of the first cohort, based on modified USPHS criteria, are presented in Table 4. An exemplary case is depicted in Figure 2.

At the 10.7-year follow-up, none of the restorations showed secondary caries, and all teeth were therefore rated ‘Alpha’. Likewise, none of the teeth showed any signs of hypersensitivity when provoked with air (100% ‘Alpha’ ratings). Highly predominant ‘Alpha’ ratings of the restorations were also given for the items color stability (96%), marginal inflammation (86%), and color match (77%). Marginal integrity was rated ‘Alpha’ in 55% and ‘Bravo’ in 43% of the restorations. One restoration showed a negative step (2%). Since the negative step was not removable with finishing, this restoration was rated ‘Charlie’. One restoration from the same patient showed localized, non-removable discoloration in the marginal area, which was rated ‘Charlie’ (2%). The other restorations received ‘Alpha’ (57%) and ‘Bravo’ (41%) ratings in terms of marginal discoloration. The item anatomical form was predominantly rated ‘Bravo’ (57%), due to visible loss of composite material as a result of attrition. Three restorations (5%) showed small composite defects extending to the tooth surface and were therefore rated ‘Charlie’. For 38% of the restorations the anatomic form was rated ‘Alpha’. ‘Alpha’ (52%) and ‘Bravo’ (48%) ratings were about equally present for the item surface texture.

During the 10.7-year follow-up period, a total of 10 restorations showed unfavorable events which could be repaired. These unfavorable events were mainly bulk fractures (*n* = 6), one secondary caries lesion, one marginal gap, one marginal discoloration, and one void within the composite material. Three restorations needed replacement and were therefore considered as failures. The reasons for failure were complete debonding (*n* = 1), and severe wear (*n* = 1) and bulk fracture (*n* = 1) which were not repairable. The success rate of the restorations was 78.0% at 10.7 years. The functional survival of the restorations was 94.9%.

### 3.2. Second (Medium-Term) Cohort

**Figure 3:**
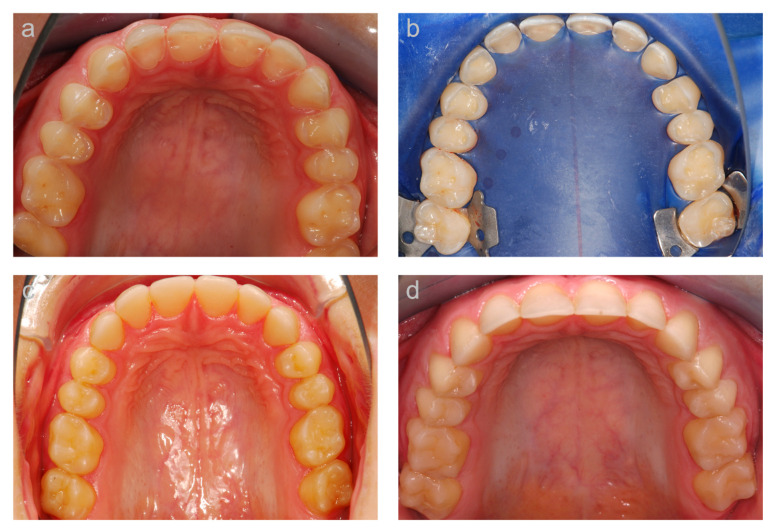
Example of a case (occlusal view of upper jaw) of the second cohort. (**a**) Baseline situation; (**b**) clinical situation at the reconstructive appointment before treatment, and (**c**) after the composite vertical bite reconstruction; (**d**) clinical situation at the 5-year follow-up.

The results of the clinical evaluation of the second cohort are presented in Table 5. An exemplary case is shown in Figure 3. At the 5.2-year follow-up, the restorations were predominantly rated ‘Alpha’ for all items, except for marginal inflammation, which showed higher ‘Bravo’ than ‘Alpha’ ratings. Not a single tooth showed signs of hypersensitivity upon provocation with air, and only one secondary caries was observed. One restoration (1%) was rated ‘Charlie’ with respect to the item margin integrity due to a non-removable negative step. Five restorations (5%) showed non-removable discoloration in the marginal area and were therefore rated ‘Charlie’ for the item marginal discoloration. With respect to the anatomical form, nine restorations (9%) were rated ‘Charlie’ since the observed loss of composite material extended to the tooth surface. For a comparison of the nanofilled composite restorations in the second cohort with the microhybrid composite restorations used in the first cohort, the results of the microhybrid composite restorations after a similar mean observation time of 5.5 years [25] are also presented (Table 5). The nanofilled composite restorations showed higher ‘Alpha’ ratings, and lower ‘Bravo’ and ‘Charlie’ ratings, for the items anatomical form, marginal integrity, and marginal discolorations. Similarly high ‘Alpha’ ratings of the nanofilled and microhybrid composite were revealed with respect to surface texture (74% and 72%, respectively). The nanofilled composite restorations showed on the other hand lower ‘Alpha’ and higher ‘Bravo’ ratings compared to the microhybrid composite restorations for the items color stability and color match.

During the 5.2-year follow-up period (second cohort), a total of 23 unfavorable, repairable events occurred. These unfavorable events were mainly bulk fractures (*n* = 14), one secondary caries lesion, one marginal gap, and seven marginal discolorations. The success rate of the restorations was 78.1% at 5.2 years. None of the restorations was lost or had to be replaced, resulting in a functional survival rate of 100%.

### 3.3. Patient Satisfaction/Compliance

The results of the subjective patient satisfaction using visual-analogue scales (VAS) are given in Table 6. In general, all patients displayed very good acceptance of the treatment. They were very satisfied with the chewing comfort and did not develop muscle or joint problems with the vertical bite reconstructions. The cost-benefit ratio was judged to be very positive, and all patients from both cohorts would recommend their treatment to other patients without hesitation. Six patients reported that they wear the night guard regularly, two patients just occasionally, and five patients never used the night guard.

## 4. Discussion

Pathological tooth wear represents a major problem for both the patient and the dentist when teeth are severely damaged and the vertical dimension of occlusion is reduced. The restorative challenge is to provide reconstructions to rehabilitate the occlusion with the least loss of dental hard tissue in dentitions that have already lost a significant amount of tooth structure due to the tooth wear process. The present investigation is the first long-term follow-up of non-invasive vertical bite reconstructions with direct composite materials. In general, we found a good overall clinical performance of the restorations after a mean observation time of 10.7 years using the USPHS criteria, and an excellent patient acceptance of the treatment. When repairs were not considered as failures, the functional survival of the restorations was excellent with 94.9% surviving restorations in place, especially when taking into account the large size of the composite build-ups. Furthermore, the success rate of 78.0% after 10.7 years is comparable to findings of clinical studies on routine posterior composite restorations placed in patients without documented tooth wear [31,32].

Our results indicate that large direct composite restorations can be used to re-establish the vertical dimension of occlusion in patients with severely eroded teeth, even in the long-term. Thus, the presented non-invasive treatment approach may help clinicians to avoid more traditional reconstructive concepts for severely worn dentitions, which are mainly based on invasive methods, such as full or partial crown coverage. In spite of the limited number of patients enrolled in the present investigation and the retrospective cohort design, which are limitations of the study, it gives an adequate impression of the success of direct composite restorations placed on increased vertical dimension discrepancies. Previous studies with observation times up to four years also found high success rates of full rehabilitations with direct composite build-ups for treatment of severe tooth wear [24,27]. On the other hand, Bartlett and Sundaram [22] reported a failure rate of 50% for composite restorations used to reconstruct posterior worn teeth. The high failure rate might be explained by the fact that the restorations were made of microfilled composites, which have been shown to possess very low physicomechanical properties, such as flexural strength [33], and might therefore be contraindicated for extensive restorations under high masticatory load.

The good clinical performance of the composite vertical bite reconstructions in the long-term cohort was confirmed in the second cohort with a functional survival of 100% at 5.2 years. All defective restorations could be repaired in this cohort. In both cohorts, the survival of the restorations could be considerably increased by repair measures, which emphasizes the importance of interventive maintenance care in the treatment of severe tooth-wear patients [11]. In the present investigation, the main reason for repairs were bulk fractures, which is in line with previous studies on composite build-up restorations in worn dentitions [24,27]. Patients with fractured composite restorations reported that they did not wear the prescribed protective splint (night guard) or did not wear it regularly. Therefore, supportive splint therapy might reduce the fracture risk of restorations, even though evidence for the effectiveness of night guards is limited [27,34].

All patients were included in a regular recall system based on their individual cariologic and periodontal risk assessment, but with a minimum of two visits per year. Special emphasis was put on their dietary and functional habits, and patients were re-instructed accordingly, if required. All patients received individualized caries prophylaxis with professional fluoridation. Restoration interfaces were thoroughly assessed during caries control; however, discolored margins were not routinely removed and polished due to the quasi-study character of the cohorts in order not to modify and alter the clinical presentation of the treated teeth in future assessments.

While in the first cohort, the direct adhesive build-up restorations were made of a fine particle microhybrid composite material (Tetric, Ivoclar Vivadent), which was state of the art as a restorative material for posterior teeth at the time the restorations were performed, in the second cohort, a nanofilled composite (Filtek Supreme, 3M) was used. The clinical evaluation using USPHS criteria revealed considerably higher ‘Alpha’ ratings, and considerably lower ‘Bravo’ ratings, of the nanofilled composite restorations in terms of anatomical form, when compared with the microhybrid composite restorations at a similar 5-year restoration service time. Both laboratory [35] and clinical studies [36] substantiated superior wear resistance of nanofiller-modified composites compared to conventional microhybrid composites. Moreover, nanofilled composite restorations have been shown to cause lower wear on the opposing tooth than microhybrid composite restorations [18]. In addition, the incorporated nanoclusters provide a distinct reinforcing mechanism compared with conventional composites providing good physicomechanical properties [37,38].

The superior clinical performance of the nanofilled composite restorations (second cohort) with respect to marginal integrity and marginal discoloration compared to the microhybrid composite restorations (first cohort) might also be due to the fact that the composite material used in the second cohort was preheated inside the template before placement. Preheating composites has been shown to reduce material viscosity [39] and decrease shrinkage forces during polymerization [30], leading to enhanced marginal adaptation [40]. Furthermore, the fact that in the second cohort, Optibond FL (Kerr) instead of Syntac Classic (Ivoclar Vivadent) was used as the adhesive system, might have affected the clinical performance and contributed to superior margin quality of the restorations [17].

## 5. Conclusions

In patients with severely worn dentitions, full rehabilitations with direct composite restorations represent a valuable long-term treatment option to re-establish the vertical dimension of occlusion with a non-invasive and relatively inexpensive technique. Vertical bite reconstructions performed with nanofilled composite show less surface degradation and better margin qualities than microhybrid composite build-up restorations.

## Figures and Tables

**Table 1:** Patients and teeth involved (first cohort).

	Gender	Patient Age (Years)	Treated Teeth	Observation Period (Years)
1	Male	45	17, 16, 15, 14, 24, 25, 26, 27, 37, 36, 35, 34, 44, 45, 47	10.6
2	Male	39	37, 36, 35, 34, 44, 45, 46, 47	11.2
3	Female	55	37, 36, 35, 34, 44, 45, 46, 47	10.8
4	Male	43	16, 15, 14, 24, 25, 26, 37, 36, 35, 34, 44, 45, 46, 47	10.2
5	Male	42	17, 16, 15, 14, 25, 26, 37, 36, 35, 34, 44, 45, 46, 47	10.8
		**45 ± 6**	***n* = 59**	**10.7 ± 0.4**

**Table 2:** Patients and teeth involved (second cohort).

	Gender	Patient Age (Years)	Treated Teeth	Observation Period (Years)
1	Female	28	36, 35, 34, 44, 45, 46	6.4
2	Male	38	17, 16, 15, 14, 24, 25, 26, 27, 37, 36, 35, 44, 45, 46, 47	7.2
3	Male	48	17, 16, 15, 14, 24, 25, 26, 27, 37, 36, 35, 34, 44, 45, 46, 47	3.9
4	Male	50	16, 15, 24, 25, 27, 36, 35, 34, 44, 45, 47	4.1
5	Male	36	17, 16, 15, 14, 24, 25, 26, 27, 37, 36, 35, 34, 44, 45, 46, 47	6.8
6	Female	27	17, 16, 15, 14, 24, 25, 26, 27, 37, 36, 35, 34, 44, 45, 46, 47	5.1
7	Male	51	17, 16, 15, 14, 24, 25, 26, 27, 37, 36, 35, 34, 44, 45, 46, 47	4.9
8	Female	56	15, 14, 24, 25, 38, 35, 34, 44, 45	3.4
		**42 ± 11**	***n* = 105**	**5.2 ± 1.4**

**Table 3:** Modified United States Public Health Service (USPHS) criteria applied for the clinical evaluation of the restorations.

	Alpha	Bravo	Charlie	Delta
Surface texture	Sound	Rough	-	-
Anatomical form	Sound	Loss of material within the composite	Loss of material extending to the tooth surface	Complete or partial (>50%) loss of the bulk
Marginal integrity	Sound	Positive/negative step, removable by finishing	Negative step, not removable by finishing	Strong negative step, not removable in major parts
Marginal discoloration	None	Slight discoloration, removable by finishing	Discoloration, localized, not removable	Strong discoloration in many parts, not removable
Secondary caries	None	-	Caries present	-
Marginal inflammation	None No pockets > 3 mm, no bleeding	Slight No pockets > 3 mm, bleeding	Moderate Pockets 4–5 mm, bleeding	Severe Pockets ≥ 6 mm, bleeding
Restoration color stability	No change	Change of color as compared to baseline	-	-
Color match	Sound	Non-perceptible at talking distance	Perceptible at talking distance	Total mismatch
Postoperative sensitivity (air)	None	Moderate	Severe	-

**Table 4:** Results of the USPHS evaluation of the restorations of the individual patients of the first study cohort still in place at the 10.7-years recall examination (number of restorations is given). The total number of teeth with the respective ratings and the percentages were calculated.

	Rating	Patient					Total	
		1	2	3	4	5	*n*	%
Surface texture	Alpha	5	2	7	5	10	**29**	**52**
	Bravo	10	6	1	7	3	**27**	**48**
	Charlie	-	-	-	-	-		
	Delta	-	-	-	-	-		
Anatomical form	Alpha	3	1	7	5	5	**21**	**38**
	Bravo	12	6	1	5	8	**32**	**57**
	Charlie	0	1	0	2	0	**3**	**5**
	Delta	0	0	0	0	0	**0**	**0**
Marginal integrity	Alpha	7	6	5	4	9	**31**	**55**
	Bravo	8	2	3	7	4	**24**	**43**
	Charlie	0	0	0	1	0	**1**	**2**
	Delta	0	0	0	0	0	**0**	**0**
Marginal discoloration	Alpha	5	7	6	3	11	**32**	**57**
	Bravo	10	1	2	8	2	**23**	**41**
	Charlie	0	0	0	1	0	**1**	**2**
	Delta	0	0	0	0	0	**0**	**0**
Secondary caries	Alpha	15	8	8	12	13	**56**	**100**
	Bravo	-	-	-	-	-		
	Charlie	0	0	0	0	0	**0**	**0**
	Delta	-	-	-	-	-		
Marginal inflammation	Alpha	15	8	5	11	9	**48**	**86**
	Bravo	0	0	3	1	4	**8**	**14**
	Charlie	0	0	0	0	0	**0**	**0**
	Delta	0	0	0	0	0	**0**	**0**
Restoration color stability	Alpha	15	7	8	12	12	**54**	**96**
	Bravo	0	1	0	0	1	**2**	**4**
	Charlie	-	-	-	-	-		
	Delta	-	-	-	-	-		
Color match	Alpha	14	7	2	11	9	**43**	**77**
	Bravo	1	1	6	1	4	**13**	**23**
	Charlie	0	0	0	0	0	**0**	**0**
	Delta	0	0	0	0	0	**0**	**0**
Postoperative sensitivity (air)	Alpha	15	8	8	12	13	**56**	**100**
	Bravo	0	0	0	0	0	**0**	**0**
	Charlie	0	0	0	0	0	**0**	**0**
	Delta	-	-	-	-	-		

**Table 5:** Results of the USPHS evaluation of the second study cohort (nanofilled composite restorations) after a mean restoration service time of 5.2 years, and of the first study cohort (microhybrid composite restorations) after a similar restoration service time *. The percentages of the respective ratings (alpha–delta) for the different categories of the clinical evaluation are given.

	Alpha		Bravo		Charlie		Delta	
	Nanofilled	Microhybrid *	Nanofilled	Microhybrid *	Nanofilled	Microhybrid *	Nanofilled	Microhybrid *
Surface texture	74	72	26	28	-	-	-	-
Anatomical form	74	29	17	57	9	13	0	0
Marginal integrity	80	41	19	57	1	5	0	1
Marginal discoloration	81	39	14	51	5	11	0	0
Secondary caries	99	100	-	-	1	0	-	-
Marginal inflammation	41	41	59	59	0	0	0	0
Restoration color stability	91	97	9	3	-	-	-	-
Color match	72	89	28	11	0	0	0	0
Postoperative sensitivity (air)	100	96	0	4	0	0	-	-

* Values taken from Attin et al. [25].

**Table 6:** Mean (±standard deviation) visual analogue scale (VAS) scores of the first (10.7-year) and second (5.2-year) cohort.

	VAS Score	
	5.2-Year Cohort	10.7-Year Cohort
Chewing comfort (0 = maximal unsatisfied, 10 = maximal satisfied)	9.7 (0.3)	8.9 (0.8)
Recommend treatment (0 = not at all, 10 = without hesitation, anytime)	9.5 (0.9)	9.7 (0.4)
Muscle or joint problems (0 = not at all, 10 = maximal)	0.8 (2.2)	1.2 (2.2)
Cost-benefit ratio (0 = maximal unsatisfied, 10 = maximal satisfied)	7.6 (2.3)	9.1 (0.9)

## Data Availability

Data is contained within the article.
